# Identification of three novel homozygous variants in *COL9A3* causing autosomal recessive Stickler syndrome

**DOI:** 10.1186/s13023-022-02244-6

**Published:** 2022-03-03

**Authors:** Aboulfazl Rad, Maryam Najafi, Fatemeh Suri, Soheila Abedini, Stephen Loum, Ehsan Ghayoor Karimiani, Narsis Daftarian, David Murphy, Mohammad Doosti, Afrooz Moghaddasi, Hamid Ahmadieh, Hamideh Sabbaghi, Mohsen Rajati, Narges Hashemi, Barbara Vona, Miriam Schmidts

**Affiliations:** 1 Department of Otolaryngology, Head and Neck Surgery, Tübingen Hearing Research Centre, Eberhard Karls University, 72076 Tübingen, Germany; 2 Genome Research Division, Human Genetics Department, Radboud University Medical Center, Geert Grooteplein Zuid 10, Nijmegen, The Netherlands; 3 Pediatric Genetics Division, Center for Pediatrics and Adolescent Medicine, University Hospital Freiburg, Freiburg University Faculty of Medicine, Mathildenstrasse 1, 79106 Freiburg, Germany; 4 Ophthalmic Research Center, Research Institute for Ophthalmology and Vision Science, Shahid Beheshti University of Medical Sciences, Tehran, Iran; 5 Department of Medical Genetics and Molecular Medicine, School of Medicine, Mashad University of Medical Science, Mashad, Iran; 6 Department of Molecular Genetics, Next Generation Genetic Polyclinic, Mashhad, Iran; 7 Ocular Tissue Engineering Research Center, Research Institute for Ophthalmology and Vision Science, Shahid Beheshti University of Medical Sciences, Tehran, Iran; 8 Ophthalmic Epidemiology Research Center, Research Institute for Ophthalmology and Vision Science, Shahid Beheshti University of Medical Sciences, Tehran, Iran; 9 Department of Otorhinolaryngology, School of Medicine, Ghaem Hospital, Sinus and Surgical Endoscopic Research Center, Mashhad University of Medical Sciences, Mashhad, Iran; 10 Department of Pediatric Neurology, Faculty of Medicine, Mashhad University of Medical Sciences, Mashhad, Iran; 11 Present Address: Institute of Human Genetics, University Medical Center Göttingen, Göttingen, Germany; 12 Present Address: Institute for Auditory Neuroscience and InnerEar Lab, University Medical Center Göttingen, Göttingen, Germany; 13 CIBSS - Centre for Integrative Biological Signalling Studies, University of Freiburg, 79104 Freiburg, Germany

**Keywords:** Autosomal recessive Stickler syndrome, COL9A3, Collagen, Hearing loss, Retinal detachment

## Abstract

**Background:**

Stickler syndrome (STL) is a rare, clinically and molecularly heterogeneous connective tissue disorder. Pathogenic variants occurring in a variety of genes cause STL, mainly inherited in an autosomal dominant fashion. Autosomal recessive STL is ultra-rare with only four families with biallelic *COL9A3* variants reported to date.

**Results:**

Here, we report three unrelated families clinically diagnosed with STL carrying different novel biallelic loss of function variants in *COL9A3*. Further, we have collected *COL9A3* genotype–phenotype associations from the literature.

**Conclusion:**

Our report substantially expands the molecular genetics and clinical basis of autosomal recessive STL and provides an overview about allelic COL9A3 disorders.

**Supplementary Information:**

The online version contains supplementary material available at 10.1186/s13023-022-02244-6.

## Background

Stickler syndrome (STL) is a rare, clinically and genetically heterogeneous connective tissue disorder divided into six clinical subtypes with overlapping features, including ocular pathologies (myopia, retinal detachment, vitreoretinal degeneration, cataract), hearing impairment (sensorineural, mixed, and/or conductive), craniofacial abnormalities (midface hypoplasia, anteverted nares, depressed nasal bridge and either Pierre Robin sequence or cleft palate and micrognathia) and joint problems (mild spondyloepiphyseal dysplasia, and precocious osteoarthritis) [1]. These features exhibit substantial variable expressivity according to clinical subtype [1]. STL is molecularly diagnosed by the presence of pathogenic variants in six collagen-type genes including *COL2A1*, *COL11A1*, *COL11A2*, *COL9A1*, *COL9A2*, *COL9A3,* and two non-collagen genes consisting of *LRP2* and *LOXL3* [1–3], following a predominantly autosomal dominant inheritance pattern.

The heteropolymer collagen XI/IX/II are critical in the extracellular matrix of joints, bones, ligaments and connective tissues throughout the body [4]. *COL2A1* encodes collagen type II alpha 1 chain. Heterozygous variants that cause functional haploinsufficiency are responsible for autosomal dominant STL type I (OMIM #108300), representing the most common subtype, accounting for roughly 80–90% of STL [5, 6]. Pathogenic variants in *COL11A1* cause the second most common STL subtype, type II (OMIM #604841) (10–20%). Variants in this gene likewise typically follow a dominant inheritance pattern [7], although five families have been described with STL and biallelic *COL11A1* mutations [8–10]. *COL11A2* pathogenic variants are very rare and cause autosomal dominant non-ocular Stickler syndrome (type III, OMIM#184840), also known as otospondylomegaepiphyseal dysplasia (OSMEDA, OMIM# 120290), as well as Weissenbacher-Zweymuller syndrome (WZS) (OMIM #184840) [11]. Biallelic variants in *LOXL3*, a member of the lysyl oxidase family of genes, have recently been causally associated with STL in two unrelated families [2, 12]. A biallelic missense variant in *LRP2* has likewise been suggested to cause STL [3].

Collagen IX proteins are encoded by *COL9A1*, *COL9A2* and *COL9A3* that together form fibril heterotrimer associated collagens and have been recently linked to autosomal recessive STL [13]. Very recently, heterozygous *COL9A3* variants have been identified as causing peripheral vitreoretinal degeneration and retinal detachment [14]. *COL9A1* and *COL9A2* are causally associated with autosomal recessive STL type IV (OMIM #614134) and V (OMIM #61484), respectively. The main clinical characteristics of individuals affected with biallelic *COL9A1* variants include moderate-to-severe sensorineural hearing loss, moderate-to-high myopia with vitreoretinopathy, and epiphyseal dysplasia, whereas *COL9A2* variants are associated with high myopia, vitreoretinal degeneration, retinal detachment, hearing loss, and short stature. Only very recently, biallelic mutations in *COL9A3* have been described to cause autosomal recessive STL in four unrelated families with seven patients. The main phenotypes that are common in all these patients consisted of high myopia, moderate to severe sensorineural hearing loss, and spondylo/epiphyseal dysplasia. Here, we report three additional unrelated consanguineous STL families with five affected individuals in total who each present three novel biallelic *COL9A3* variants.

## Results

### Clinical assessments

**Fig. 1:**
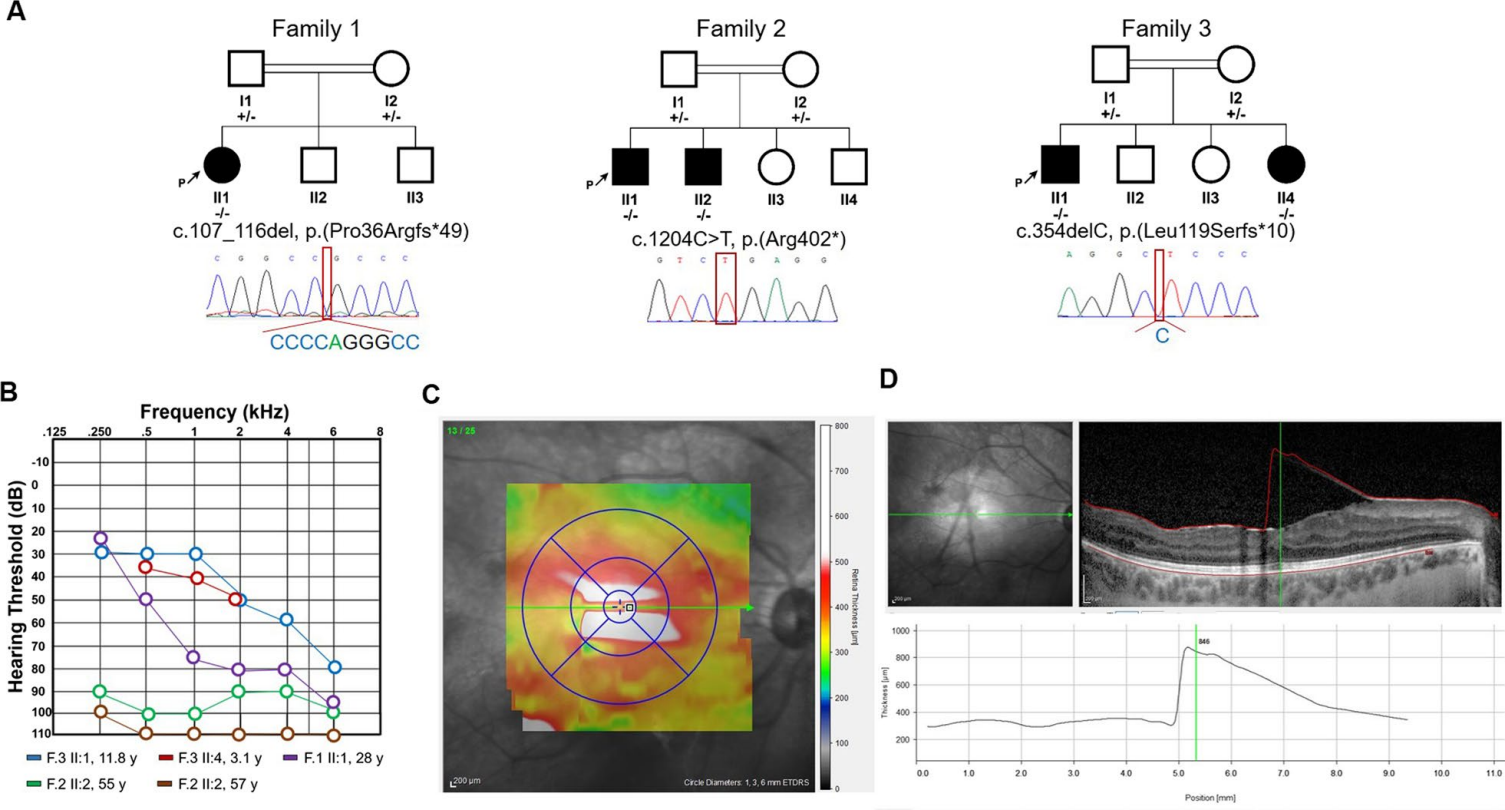
**A** Pedigrees and electropherogram of affected individuals with biallelic *COL9A3* variants. **B** Audiograms of right ears of all affected individuals from three families. **C**, **D** Optical coherence tomography (OCT) imaging in proband II1 from family 1 showing retinal detachment

**Fig. 2:**
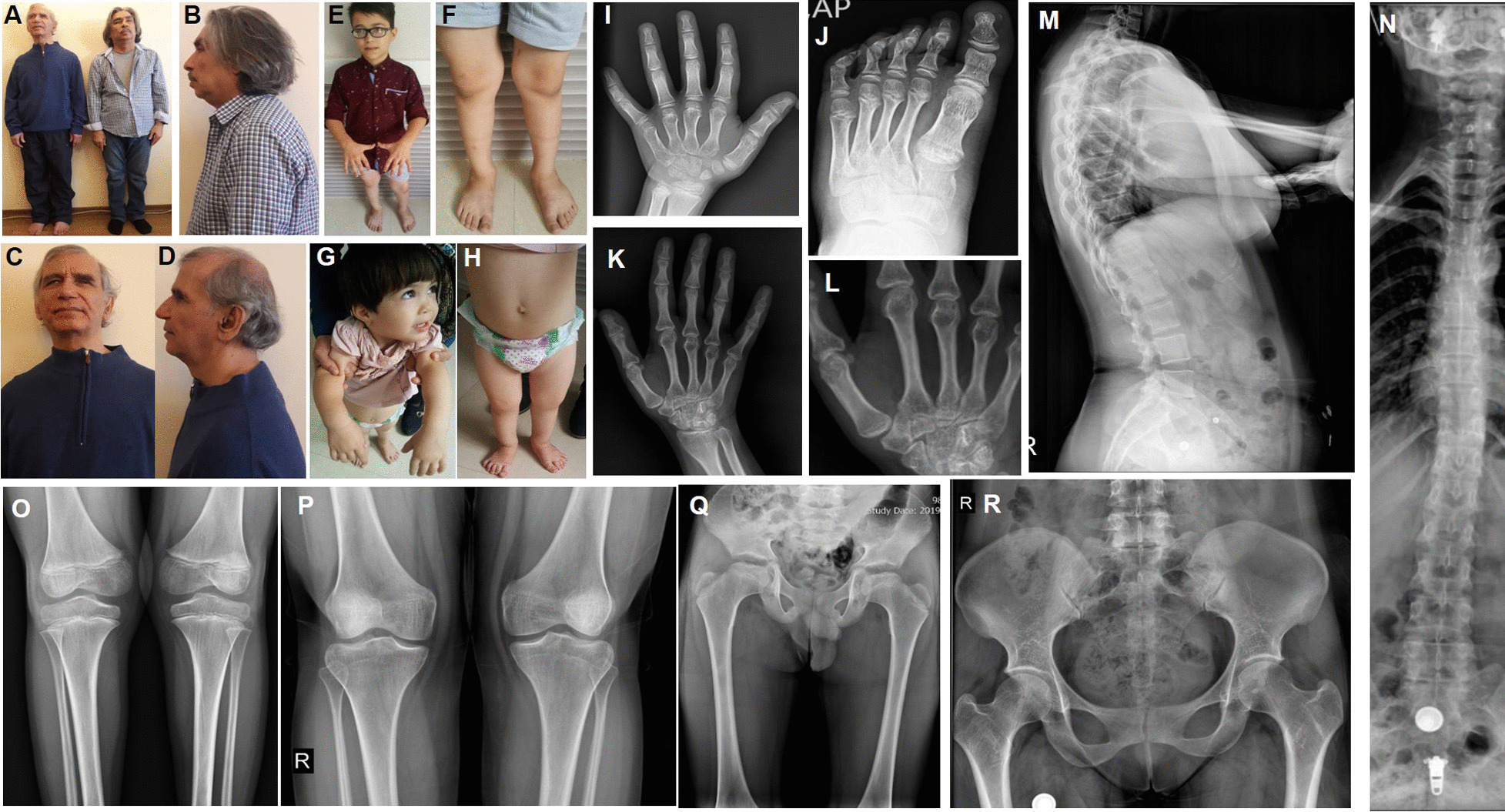
Pictures of four affected individuals and standard radiographs of the spine, pelvis and limbs of two patients. **A**–**D** Pictures of two affected individuals from family 2 who both have herniated cervical discs. **E**, **F** Pictures of affected individual II1 from family 3 showing short stature, pes planus, bowed tibia, genu valga and rotated distal femura distal femur. **G**, **H** Clinical appearance of proband II1 from family 1 showing pes planus, mild midface hypoplasia, upturned nose and low set ears. **I**, **J** Hand and foot radiographs showing short metacarpalia and a broad big toe for individual II1 from family 3 at the age of 10 years. **K**, **L** Radiograph images of the right hand and wrist joint of individual II1 from family 1 at the age of 28 years, showing short metacarpalia with widened epiphyses and an irregular radius epiphysis. **M**, **N** Radiographs of the spine of II1 from family 1, showing mild platyspondyly of the thoracic spine as well as signs of ankylosing spondylitis. **O** Knee radiograph of proband II1 from family 3 showing genua valga and irregular femur epiphyses. **P** Knee radiograph of proband II1 from family 1 demonstrating genua valga and widened femur epiphyses. **Q** Pelvis radiograph individual II1 of from family 3 showing a flat acetabular roof with irregularities and flattened capiti femori, as well as broadened and shortened necks. **R** Radiograph of the pelvis of individual II1 from family 1 showing a relatively narrow intraarticular space but well developed capiti femori and no flattening of the acetabular roof

Three unrelated consanguineous families of Iranian descent were referred for genetic testing due to hearing and vision impairment (Fig. 1), as well as skeletal dysplasia that resulted in a clinical diagnosis of STL (Fig. 2).

The female proband (II1) from Family 1 is the oldest and only affected individual out of three children from first cousin parents. She had a normal delivery and birth, with a birth weight of 3.2 kg (− 0.43 SD). She was 28 years old at last examination with a weight of 64 kg (+ 0.41 SD), height of 157 cm (− 0.8 SD) and occipitofrontal circumference (OFC) of 55 cm (+ 0.62 SD). She suffers from high myopia in both eyes, in addition to vitreoretinal degeneration with empty vitreous, multiple lattice degenerations and retinal pigmentary changes. There was unilateral absence of the frontal sinus in her skull X-ray. She has severe and progressive sensorineural hearing loss. X-ray and detailed examination of her joints and bones, including mobility testing and examination for signs of osteoarthritis were normal, however she complained of pain in her knee joints. Typical STL craniofacial features such as midface hypoplasia, cleft palate, micrognathia, depressed nasal bridge and anteverted nares are absent.

Family 2 presented with two affected individuals out of four children who were born from a first cousin marriage. The proband (II1) and his affected sibling (II2) both had a normal delivery around term, measurements at birth could not be obtained. Weight, height and OFC at last clinical assessment (at 65 and 57 years-old) were 68 kg (− 0.16 SD) and 66 kg (− 0.39 SD), 166 cm (− 1.4 SD) and 163 cm (− 1.8 SD), 56 cm (+ 0.62 SD) and 57 cm (+ 1.32 SD), respectively. Both had a history of multiple vitreoretinal surgeries due to recurrent rhegmatogenous retinal detachments resulting from advanced vitreoretinal degeneration. Despite vitreoretinal surgeries, the older patient is considered blind without light perception (NLP) in either eye while his sibling has counting finger vision for one eye while NLP was noted for the other eye. Both suffer from severe and progressive sensorineural hearing loss. Likewise, both show a herniated cervical disc and muscular atrophy was noted in the older sibling. No radiologic documentation was available for review.

Family 3 presented with two affected and two healthy children from first cousin parents. Both affected individuals had normal delivery with a birth weight of 3.4 kg (− 0.26 SD) and 3.75 kg (+ 0.71 SD), length of 49 cm (− 0.6 SD) and 49.5 cm (0.1 SD), and OFC of 35 cm (− 0.40 SD) and 36 cm (+ 0.62 SD). The most current weight, height and OFC measurements for the proband (II1) at age 11.8 years and his sister (II4) at age 3.1 years are 32 kg (-1.43 SD), 137 cm (0.9 SD), and 53 cm (− 0.53 SD) and 12 kg (− 0.05 SD), 84 cm (− 0.6 SD), and 48 cm (+ 0.39 SD), respectively. Both affected individuals have myopia and congenital moderate to severe progressive sensorineural hearing impairment. The affected male complains of knee joint pain, especially when he runs. X-ray and detailed examination demonstrated spondyloepiphyseal dysplasia in both children. Both individuals II1 and II4 have pes planus, depressed nasal bridge and anteverted nares, with midface hypoplasia and downslanting palpebral fissures more pronounced in II4. Detailed clinical features of all affected individuals are described in Table 1 and Additional file 1: Table S1. None of the individuals showed signs of intellectual disability.

**Table 1:** Summary of genetic and clinical findings in probands with biallelic *COL9A3* variants

	p.(Pro36Argfs*49) Family 1	p.(Arg402*) Family 2, Patient1	p.(Arg402*) Family 2, Patient 2	p.(Leu119Serfs*10) Family 3, Patient 1	p.(Leu119Serfs*10) Family3, Patient 2	p.(Gln393Cysfs*25) Faletra et al. [15] Patient 1
Ethnicity	Iranian	Iranian	Iranian	Iranian	Iranian	Moroccan
Consanguinity	First cousin	First cousin	First cousin	First cousin	First cousin	First cousin
Sex	Female	Male	Male	Male	Female	Female
Age in years	28	65	57	11 years, 8 months	3 years,1 month	4
Birth	Uncomplicated (normal delivery)	Uncomplicated (normal delivery)	Uncomplicated (normal delivery)	Uncomplicated	Uncomplicated	NA
*Measurements*						
OFC at last examination	55 cm (+ 0.62 SD)	56 cm (+ 0.62 SD)	57 cm (+ 1.32 SD)	53 cm (− 0.53 SD)	48 cm (+ 0.39 SD)	NA
Weight at last evaluation	64 kg (+ 0.41 SD)	68 kg (− 0.16 SD)	66 kg (− 0.39 SD)	32 kg (− 1.43 SD)	12 kg (− 0.05 SD)	16 kg
Height at last examination	157 cm (− 0.8 SD)	166 cm (− 1.4 SD)	163 cm (− 1.8 SD)	137 cm (0.9 SD)	84 cm (− 0.6 SD)	107 cm
Myopia	Moderate-to-high	High	High	High	High	Moderate-to-high
Vitreoretinal degeneration	No	Yes	Yes	No	No	No
Cataract	No	Yes	Yes	No	No	No
Retinal detachment	No	Yes	Yes	No	No	No
*Auditory system*						
Hearing loss	Yes	Yes	Yes	Yes	Yes	Yes
Age at onset	NA	NA	NA	Early onset	Early onset	Early onset
Type	Sensorineural	Sensorineural	Sensorineural	Sensorineural	Sensorineural	Sensorineural
Degree of hearing loss	Severe	Profound	Profound	Moderate-to-severe	Moderate-to-severe	Moderate-to-severe
Progressive/stable	Progressive	Progressive	Progressive	Progressive	Progressive	Progressive
*Joints*						
Short stature	No	No	No	No	No	No
Spondyloepiphyseal dysplasia	No	No	No	Yes	Yes	No
Epiphyseal dysplasia	No	No	No	Yes	Yes	Yes
*Craniofacial structures*						
Midface hypoplasia	No	No	No	No	Yes	Yes
Cleft palate	No	No	No	No	No	No

	p.(Gln393Cysfs*25) Faletra et al. [15] Patient 2	p.(Gln393Cysfs*25) Faletra et al. [15] Patient 3	p.(Pro218Alafs*49) Hanson-Kahn et al. [16]	p.(Arg471Ter) Nixon et al. [13] Patient 1	p.(Arg471Ter) Nixon et al. [13] Patient 2	p.(Arg90Ter) and p.(Arg577Ter) Markova et al. [19]
Ethnicity	Moroccan	Moroccan	Indian	NA	NA	Russian
Consanguinity	First cousin	First cousin	Third cousin	NA	NA	No
Sex	Male	Male	NA	NA	NA	Male
Age in years	11	16	12	18	20	
Birth	NA	NA	Uncomplicated (Caesarean section)	NA	NA	At term
*Measurements*						
OFC at last examination	NA	NA	NA	NA	NA	NA
Weight at last evaluation	38 kg	60 kg	NA	NA	NA	13 kg (50th %ile)
Height at last examination	144 cm	170 cm	NA	NA	NA	88 cm (25–50th %ile)
Myopia	Moderate-to-high	Moderate-to-high	High	High	High	High
Vitreoretinal degeneration	No	No	No	No	No	Yes
Cataract	No	No	No	No	No	No
Retinal detachment	No	No	No	No	No	No
*Auditory system*						
Hearing loss	Yes	Yes	Yes	Yes	Yes	Yes
Age at onset	NA	NA	Early onset	NA	NA	Yes
Type	Sensorineural	Sensorineural	Sensorineural	Sensorineural	Sensorineural	Senorineural
Degree of hearing loss	Moderate-to-severe	Moderate-to-severe	Moderate-to-severe	Severe	Severe	Severe
Progressive/stable	Progressive	Progressive	Stable	Progressive	Progressive	NA
*Joints*						
Short stature	No	No	No	No	No	No
Spondyloepiphyseal dysplasia	No	No	No	No	No	Yes
Epiphyseal dysplasia	Yes	Yes	Yes	NA	NA	Yes
*Craniofacial structures*						
Midface hypoplasia	Yes	Yes	Yes	No	No	Yes
Cleft palate	No	No	No	No	No	No

*NA* not ascertained, *OFC* occipitofrontal circumference, *SD* standard deviation

### Genetic analysis

The DNA of probands from the three unrelated families (family 1 Proband II1, family 2 proband II1, family 3 proband II1) was subjected to Exome Sequencing (ES), revealing three different novel, homozygous loss of function (LOF) variants in *COL9A3*, NM_001853.3. The proband in Family 1 was found to have a *COL9A3* deletion (c.107_116del, p.(Pro36Argfs*49), rs1470627424), causing a frameshift in exon 2. The allele frequency in gnomAD is 0.00001390 with two carriers, while other public genomic databases such as Iranome and GME, and 1000 genomes have not reported this variant. The proband in family 2 disclosed a *COL9A3* nonsense variant (c.1204C > T, p.(Arg402*), rs989413835) in exon 23, while the proband in Family 3 showed a one base pair deletion in *COL9A3* [c.355delC, p.(Leu119Serfs*9)] in exon 7. Both variants have not been reported in public databases.

## Discussion

**Fig. 3:**
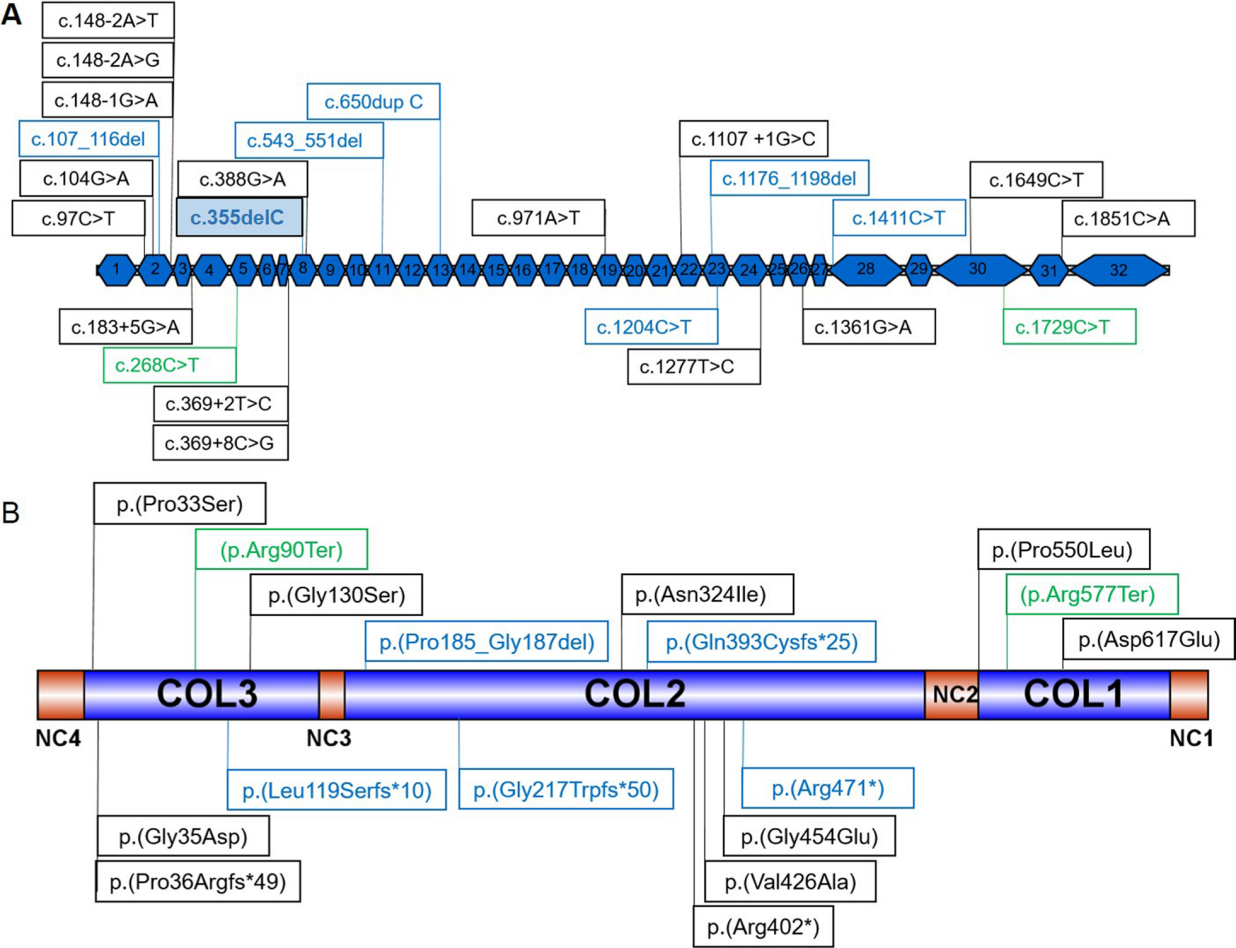
Overview of known and novel variants in COL9A3 at the cDNA and protein levels. All variants have been reported by HGMD Professional 2020.1.and classified as pathogenic in ClinVar or are reported but have not yet been classified in ClinVar. The black words indicate heterozygous variants, the blue words show homozygous variants and the green words show the heterozygous compound variants. **A** Variants on cDNA level; **B** variants on protein level

Here, we report three families with five affected individuals clinically diagnosed with autosomal recessive STL due to biallelic LOF variants in *COL9A3*. Our report re-affirms previous studies that have described four families with biallelic LOF causing autosomal recessive STL, increasing the total number of families reported to date to seven [13, 15, 16]. These COL9A3 variants as well a other disease causing COL9A3 variants submitted to HGMD are visualized in Fig. 3 for localization on cDNA as well as on protein level.

*COL9A3*, along with two other heterodimers (*COL9A1* and *COL9A2*), belongs to the collagen IX complex, forming a fibril-associated collagen with interrupted triple (FACIT) helices and connecting with collagen II and XI fibrils. A *Col9a1* knockout mouse study previously demonstrated that absence of this protein in mice results in the loss of the entire collagen IX heterotrimer complex [17]. Recent reports on the clinical phenotype of STL and MED syndromes that are caused by variants affecting different members of collagen IX have supported the hypothesis that each of the three proteins is essential for collagen IX function [13, 18].

While a variety of disorders have been described to result from heterozygous pathogenic variants in *COL9A3*, only four unrelated STL families and one family with nonsyndromic hearing loss have been reported to date carrying biallelic variants (Table 2). Allelic disorders resulting from *COL9A3* variants include nonsyndromic hearing loss, MED, pseudoachondroplasia, cerebral palsy, and lumbar disc disease and severe peripheral vitreoretinal degeneration and retinal detachment (Table 2).

**Table 2:** Pathogenic *COL9A3* variants reported in HGMD and associated clinical phenotypes

c.DNA position	Protein position	Exon/intron	Description	Zygosity	dbSNP	ClinVar	Reported phenotype	References
–	–		99 bp duplication (CNV)	Het	NA	NA	Sensorineural hearing loss	Ji (2014) BMC Ear Nose Throat Disord 14,9
c.97C > T	p.(Pro33Ser)	2	Missense	Het	rs745914662	NA	Cerebral palsy	Pingel (2019) Am J Med Genet B Neuropsychiatr Genet 180,12
c.104G > A	p.(Gly35Asp)	2	Missense	Het	rs1390736361	NA	Multiple epiphyseal dysplasia	Jeong (2014) BMC Musculoskelet Disord 15,371
c.148-1G > A	p.?	2	Splicing	Het	rs606231367	NA	Multiple epiphyseal dysplasia	Lohiniva (2000) Am J Med Genet 90,216
c.148-2A > G	p.?	2	Splicing	Het	NA	NA	Multiple epiphyseal dysplasia	Jackson (2012) Hum Mutat 33,144
c.148-2A > T	p.?	2	Splicing	Het	NA	P	Multiple epiphyseal dysplasia	Paassilta (1999) Am J Hum Genet 64,1036
c.183 + 5G > A	p.?	3	Splicing	Het	NA	P	Multiple epiphyseal dysplasia	Nakashima (2005) Am J Med Genet 132A,181
c.268C > T	p.(Arg90Ter)	5	Nonsense	Comp het	rs763259234	NA	Stickler syndrome	Markova (2021) Mol Genet Genomic Med
c.369 + 2T > C	p.?	7	Splicing	Het	rs1057518693	P	Multiple epiphyseal dysplasia	Posey (2017) N Engl J Med 376,21
c.369 + 8C > G	p.?	7	Splicing	Het	NA	NA	Multiple epiphyseal dysplasia	Lord (2019) Genome Res 29,159
c.388G > A	p.(Gly130Ser)	8	Missense	Het	rs139401633	VUS	Severe peripheral vitreoretinal degeneration and retinal detachment	M. Nash (2021) European Journal of Human Genetics
c.543_551del	p.(Pro185_Gly187del)	11	In frame	Hom	rs765392378	NA	Nonsyndromic hearing loss	Asamura (2005) Auris Nasus Larynx 32,113
c.650dup C	p.(Gly217Trpfster50)	13	Frameshift	Hom	NA	NA	Stickler syndrome	Hanson-Kahn (2018) Am J Med Genet A 176,2887
c.971A > T	p.(Asn324Ile)	19	Missense	Het	NA	NA	Pseudoachondroplasia	Jung (2010) Int J Mol Med 26,885
c.1107 + 1G > C	p.?	21	Splicing	Het			Severe peripheral vitreoretinal degeneration and retinal detachment	M. Nash (2021) European Journal of Human Genetics
c.1176_1198del	p.(Gln393Cyster*25)	23	Frameshift	Hom	rs606231470	VUS	Stickler syndrome	Faletra (2014) Am J Med Genet A 164,42
c.1277T > C	p.(Val426Ala)	24	Missense	Het	NA	NA	Pseudoachondroplasia	Jung (2010) Int J Mol Med 26,885
c.1361G > A	p.(Gly454Glu)	26	Missense	Het	NA	NA	Nonsyndromic hearing loss	Miyagawa (2013) PLoS One 8,e71381
c.1411C > T	p.(Arg471ter)	28	Nonsense	Hom	rs747896279	P	Stickler syndrome	Nixon (2019) Am J Med Genet A 179,1498
c.1649C > T	p.(Pro550Leu)	30	Missense	Het	rs535230112	NA	Nonsyndromic hearing loss	Miyagawa (2013) PLoS One 8, e71381
c.1729C > T	(p.Arg577Ter)	30	Nonsense	Comp het	rs1201247953	NA	Stickler syndrome	Markova (2021) Mol Genet Genomic Med
c.1851C > A	p.(Asp617Glu)	31	Missense	Het	rs199577452	NA	Nonsyndromic hearing loss	Asamura N Auris Nasus Larynx 32,113

All variants are reported using the NM_001853.3 transcript

*Comp het* compound heterozygous, *Het* heterozygous, *Hom* homozygous, *P* pathogenic, *VUS* variant of uncertain significance, *NA* not ascertained

Consistent clinical features among STL patients with biallelic *COL9A3* LOF alleles comprise moderate-to-profound progressive sensorineural hearing loss and moderate high myopia with vitreoretinal degeneration. Retinal detachment and cataract occur occasionally. In contrast, skeletal involvement seems to be more variable. For instance, Nixon et al. [13] reported a family with two affected siblings where the oldest affected sibling had severe arthropathy in the shoulders and hip, requiring a wheelchair. The X-ray of this patient showed spinal scoliosis and narrowing of the articular space in both knees, while the younger affected sibling did not show any of these signs. In line with this report, we also observed that the affected individuals in family 2, at the ages of 65 and 57 years-old, suffer only from myopia, hearing loss and each have a herniated cervical disc while the two much younger affected individuals in family 3, at ages 3 and 11 years-old, have more prominent skeletal findings that include radiological signs of spondyloepiphyseal dysplasia as well as craniofacial abnormalities including depressed nasal bridge and anteverted nares (Table 1). Moreover, Nixon et al. [13] observed that carrier parents can manifest mild STL phenotypes, while our report and others [15, 16, 19] have not observed mild phenotypes in heterozygous individuals. Besides Nixon’s report, Markova et al. [19] introduced a more severe case with compound heterozygous variants with vitreoretinal degeneration, early onset osteoarthritis, midface hypoplasia, hip dysplasia, speech developmental delay, spina bifida, kyphosis, and eye pigment rearrangement.

## Conclusion

In summary, our report consolidates that homozygous loss of function variants in *COL9A3* cause STL (type VI). We find high myopia and moderate-severe hearing loss to be consistent features amongst all cases while skeletal findings seem more variable.

## Material and methods

### Subjects

Three unrelated Iranian families with syndromic phenotypes including hearing loss, vision impairment and skeletal dysplasia were referred for clinical genetic diagnostics. Blood samples were collected after obtaining informed consent from patients or their parents. Molecular genetic diagnostic testing was performed in Nijmegen via the Radboud innovative diagnostics programme and at the University of Tuebingen (197/2019BO01). Informed consent from the parents or legal guardians of the patients/participants was obtained for the publication of their data.

### Exome and Sanger sequencing

After extraction of DNAs from whole blood by standard protocol, proband DNA samples were subjected to exome capture using the Agilent SureSelect Human All Exon V6 Kit and exome sequencing (ES) was performed on an Illumina HiSeq 2500 sequencer for an average 50 × sequencing depth, resulting in sequences of greater than 100 bases from each end of the fragments [Cambridge (Novogene UK)]. Exome data were processed for analysis using a GATK-based pipeline [20] that uses Burrows-Wheeler alignment [21] to the GRCh37/UCSC hg19 (Families 1 and 2) and GRCh38/UCSC hg38 (Family 3). VarScan version 2.2.5, MuTec and GATK Somatic Indel Detector were used to detect SNV and InDels, respectively. The protocol to interpret potential pathogenic variants was previously described [22]. For population-specific filtering, gnomAD [23], Iranome [24] and Greater Middle East (GME) Variome Project [25] databases were used.

Segregation analysis using Sanger sequencing was performed in available family members to confirm variant segregation after PCR amplification. Primers are available upon request.

## Web resources

ClinVar, https://www.ncbi.nlm.nih.gov/clinvar/.

Exome Aggregation Consortium (ExAC), http://exac.broadinstitute.org.

Genome Aggregation Database (*gnomAD*), http://gnomad.broadinstitute.org/.

## Supplementary Information

**Additional file 1**. **Table S1:** Clinical features of probands affected by COL9A3 variants. 

## Data Availability

Data can be made available on personal request. Variants reported in this study have been deposited in the Leiden Open Variation Database (LOVD) and are available through the following variant accession numbers: 0000364418, 0000364419 and 0000364420.

## Abbreviations

DNA: Deoxyribonucleic acid
ES: Exome sequencing
FACIT: Fibril-associated collagen with interrupted triple helicies
GATK: Genome Analysis Toolkit
GME: Greater Middle East
LOF: Loss of function
MED: Multiple Epiphyseal Dysplasia
OFC: Occipitofrontal circumference
PCR: Polymerase Chain Reaction
SD: Standard deviation
SNV: Single Nucleotide Variant
STL: Stickler syndrome
