# Supplementary material for: Identification of three novel homozygous variants in COL9A3 causing autosomal recessive Stickler syndrome
Source: Orphanet J Rare Dis. 2022 Mar 3;17:97. doi: 10.1186/s13023-022-02244-6 (PMC8892745; doi:10.1186/s13023-022-02244-6)
Supplement: Supplementary file 1 — Additional file 1. Table S1: Clinical features of probands affected by COL9A3 variants. [file 13023_2022_2244_MOESM1_ESM.docx]

|  | **Table S1: Clinical features of probands affected by *COL9A3* variants** | | | | | | | | | | | |
| --- | --- | --- | --- | --- | --- | --- | --- | --- | --- | --- | --- | --- |
| Variant | p.(Pro36Argfs*49) Family 1 | p.(Arg402*) Family 2, Patient1 | p.(Arg402*) Family 2, Patient 2 | p.(Leu119Serfs*10) Family 3, Patient 1 | p.(Leu119Serfs*10) Family3, Patient 2 | p.(Gln393Cysfs*25) Faletra et al.(2014) Patient 1 | p.(Gln393Cysfs*25) Faletra et al.(2014) Patient 2 | p.(Gln393Cysfs*25) Faletra et al.(2014) Patient 3 | p.(Pro218Alafs*49) Hanson-Kahn et al.(2018) | p.(Arg471Ter) Nixon et al. (2019) Patient 1 | p.(Arg471Ter) Nixon et al. (2019) Patient 2 | p.(Arg90Ter) and p.(Arg577Ter) Markova et al. 2021 |
| Ethnicity | Iranian | Iranian | Iranian | Iranian | Iranian | Moroccan | Moroccan | Moroccan | Indian | NA | NA | Russian |
| Parental consanguinity | First cousin | First cousin | First cousin | First cousin | First cousin | First cousin | First cousin | Frst cousin | Third cousin | NA | NA | No |
| Sex (year) | Female | Male | Male | Male | Female | Female | Male | Male | NA | NA | NA | Male |
| Age | 28 | 57 | 55 | 11y, 8m | 3y,1m | 4 | 11 | 16 | 12 | 18 | 20 |  |
| Birth | Uncomplicated (Normal Delivery) | Uncomplicated (Normal Delivery) | Uncomplicated (Normal Delivery) | Uncomplicated | Uncomplicated | NA | NA | NA | Uncomplicated (Caesarean section) | NA | NA | At term |
| Measurements | Measurements | | | | | | | | | | | |
| Height at birth | NA | NA | NA | 49 cm (-0.6 SD) | 49.5 cm (0.1 SD) | NA | NA | NA | 48.26 cm (50–75th %ile) | NA | NA | 51 cm |
| Weight at birth | 3200 gr (-0.43 SD) | NA | NA | 3400 gr (-0.26 SD) | 3750 gr (+0.71 SD) | NA | NA | NA | 3.32 kg (75–90th %ile) | NA | NA | 3260 gr (-0.4SD) |
| OFC at birth | NA | NA | NA | 35 cm (-0.40 SD) | 36 cm (+0.62 SD) | NA | NA | NA | NA | NA | NA | NA |
| OFC at last examination | 55 cm (+0.62 SD) | 56 cm (+0.62 SD) | 57cm (+1.32 SD) | 53 cm (-0.53 SD) | 48 cm (+0.39 SD) | NA | NA | NA | NA | NA | NA | NA |
| Weight at last evaluation | 64 kg (+0.41 SD) | 68 kg (-0.16 SD) | 66 kg (-0.39 SD) | 32 kg (-1.43 SD) | 12 kg (-0.05 SD) | 16 kg | 38 kg | 60 kg | NA | NA | NA | 13 kg (50th %il) |
| Height at last examination | 157 cm (-0.8 SD) | 166 cm (-1.4 SD) | 163 cm (-1.8 SD) | 137 cm (0.9 SD) | 84 cm (-0.6 SD) | 107 cm | 144 cm | 170 cm | NA | NA | NA | 88 cm (25-50th %il ) |
| Visual system | | | | | | | | | | | | |
| Myopia | Moderate-to-high | High | High | High | High | Moderate-to-high | Moderate-to-high | Moderate-to-high | High | High | High | High |
| Vitreoretinal degeneration | No | Yes | Yes | No | No | No | No | No | No | No | No | Yes |
| Cataract | No | Yes | Yes | No | No | No | No | No | No | No | No | No |
| Retinal detachment | No | Yes | Yes | No | No | No | No | No | No | No | No | No |
| Auditory system | | | | | | | | | | | | |
| Hearing loss | Yes | Yes | Yes | Yes | Yes | Yes | Yes | Yes | Yes | Yes | Yes | Yes |
| Age at hearing loss onset | NA | NA | NA | Early onset | Early onset | Early onset | NA | NA | Early onset | NA | NA | Yes |
| Sensorineural, mixed, and/or conductive | Sensorineural | Sensorineural | Sensorineural | Sensorineural | Sensorineural | Sensorineural | Sensorineural | Sensorineural | Sensorineural | Sensorineural | Sensorineural | Senorineural |
| Degree of hearing loss | Severe | Profound | Profound | Moderate-to-severe | Moderate-to-severe | Moderate-to-severe | Moderate-to-severe | Moderate-to-severe | Moderate-to-severe | Severe | Severe | Severe |
| Progressive or stable hearing loss | Progressive | Progressive | Progressive | Progressive | Progressive | Progressive | Progressive | Progressive | Stable | Progressive | Progressive | NA |
| Joints | | | | | | | | | | | | |
| Hypermobility | No | No | No | No | No | No | No | No | No | No | No | No |
| Precocious osteoarthritis | No | No | No | No | No | No | No | No | No | No | Yes | Yes |
| Short stature | No | No | No | Yes | Yes | No | No | No | No | No | No | No |
| Spondyloepiphyseal dysplasia | No | No | No | Yes | Yes | No | No | No | No | No | No | Yes |
| Epiphyseal dysplasia | No | No | No | Yes | Yes | Yes | Yes | Yes | Yes | NA | NA | Yes |
| Early-onset osteoarthritis | No | No | No | No | No | No | No | No | No | No | No | Yes |
| Joint pain | Yes | No | No | Yes | Yes | NA | NA | NA | NA | NA | NA | Yes |
| Craniofacial structures | | | | | | | | | | | | |
| Midface hypoplasia | No | No | No | No | Yes | Yes | Yes | Yes | Yes | No | No | Yes |
| Depressed nasal bridge | No | No | No | Yes | Yes | Yes | Yes | No | No | No | No | No |
| Anteverted nares | No | No | No | Yes | Yes | No | No | No | No | No | No | No |
| Bifid uvula | No | No | No | No | No | No | No | No | No | No | No | No |
| Cleft palate/Pierre Robin sequence | No | No | No | No | No | No | No | No | No | No | No | No |
| Micrognathia | No | No | No | No | No | No | No | No | No | No | No | No |
| Other anomalies | Unilateral absence of the frontal sinus (Left side) | Herniated cervical disc | Herniated cervical disc | Short stature, pes planus | Pes planus, palpebral fissure | Astigmatism and amblyopia due to impairment of ocular motility, Internal tibial rotation, pes planus, moderate to severe ID | Astigmatism and amblyopia due to impairment of ocular motility, Internal tibial rotation, pes planus, downslanted palpebral fissures, severe bilateral flat feet with valgus hindfoot, moderate to severe ID | Astigmatism and amblyopia due to impairment of ocular motility, Internal tibial rotation, pes planus, downslanted palpebral fissures, ptosis, moderate to severe ID | Tibial bowing, mild platyspondyly with irregularities of the vertebral endplates, autoimmune hypothyroidism |  | Severe arthropathy in shoulders and hip, mobilized in wheelchair. Spinal scoliosis surgery. Narrow intraarticular space of the knees | Hip dysplasia, speech developmental delay, spina bifida and kyphosis, eye pigment rearrangement, |
